# Diabetes Risks of Statin Therapy—Coenzyme Q10 May Help

**DOI:** 10.31083/RCM26437

**Published:** 2025-02-19

**Authors:** Xiaorong Han, Jinxing Liu, Yingzhen Gu, Yifan Li, Wei Zhang, Naqiang Lv, Aimin Dang

**Affiliations:** ^1^Department of Cardiology, Fuwai Hospital, National Center for Cardiovascular Diseases, Chinese Academy of Medical Sciences and Peking Union Medical College, 100037 Beijing, China

**Keywords:** coenzyme Q10, CoQ10, statin, new-onset diabetes

## Abstract

**Background:**

Statin therapy is associated with an increased risk of new-onset diabetes (NOD), possibly due to a reduction in coenzyme Q10 (CoQ10) levels as a result of statin use. This study aimed to investigate the relationship between exogenous CoQ10 supplementation and the development of NOD.

**Methods:**

This study included 4394 participants from the National Health and Nutrition Examination Survey (NHANES). Baseline characteristics were compared between those with and without NOD and between those with and without CoQ10. Univariate logistic regression was performed to identify factors associated with NOD. Two models were used for confounding factors, including demographics and various covariates. Multifactor logistic regression further assessed the association between CoQ10 supplementation and NOD. Additionally, restricted cubic spline (RCS) analysis was conducted to evaluate the potential nonlinear relationship between daily CoQ10 dose and NOD.

**Results:**

Univariate logistic regression showed an association between CoQ10 supplementation and a reduced risk of NOD (odds ratio [OR] = 0.323, 95% confidence interval [CI] 0.157–0.668, *p* = 0.003), which remained significant after adjustments in model 1 (OR = 0.344, 95% CI 0.160–0.737, *p* = 0.006) and model 2 (OR = 0.232, 95% CI 0.057–0.942, *p* = 0.041). There was no evidence of a linear association between daily CoQ10 dose and NOD in logistic regression analysis (OR = 0.999, 95% CI 0.994–1.004, *p* = 0.720), and no evidence of a nonlinear correlation in the RCS analysis (*p* > 0.05).

**Conclusions:**

CoQ10 supplementation in individuals taking statins was associated with a reduced risk of NOD, and this association was independent of the CoQ10 dose.

## 1. Introduction

Cardiovascular Disease (CVD) is one of the leading causes of death and disability among adults worldwide, with hyperlipidemia playing a significant role in its development [1]. Statin therapy offers clear benefits, including reducing lipid levels, slowing the progression of atherosclerosis, decreasing cardiovascular events, and is generally regarded as safe and well-tolerated [2]. However, it is essential to recognize that numerous studies and meta-analyses have confirmed the potential risk of statin use in increasing the incidence of new-onset diabetes (NOD) [3, 4, 5, 6, 7]. While the adverse effects of statin-mediated NOD are outweighed by the benefits of statin medications, NOD may negatively impact medication adherence among statin users. The mechanism underlying this population may be related to coenzyme Q10 (CoQ10) [8].

Coenzyme Q10, also known as ubiquinone, is a lipophilic compound that functions similarly to a vitamin and is synthesized endogenously from tyrosine in the human body [9]. CoQ10 acts as a lipophilic antioxidant by neutralizing free radicals, inhibiting lipid peroxidation of biomembranes, and protecting mitochondrial proteins and DNA from oxidative damage [10, 11]. Additionally, CoQ10 binds to the inner mitochondrial membrane, participating in the electron transport chain and oxidative phosphorylation, playing a crucial role in cellular energy synthesis by generating adenosine triphosphate (ATP) [12].

However, factors such as genetics, aging, and statin therapy can decrease its physiological concentration [9, 13]. Notably, low-density lipoprotein (LDL) serves as the primary carrier of CoQ10 in circulation, and is reduced by statin therapy, which may lead to decreased circulating levels of CoQ10 [13]. Furthermore, statins inhibit the synthesis of CoQ10. Insufficient CoQ10 can induce mitochondrial oxidative stress, leading to beta-cell apoptosis, reduced insulin sensitivity, and ultimately manifesting as NOD or poor blood glucose control [9, 14, 15]. Therefore, we hypothesize that appropriate CoQ10 supplementation may help reduce the risk of NOD in individuals taking statin medications.

Previous studies have confirmed that CoQ10 is associated with a reduction of NOD in mice [16, 17], but relevant studies in human population are limited [18, 19]. This study aimed to investigate whether CoQ10 supplementation has a protective effect against new-onset diabetes in a statin-taking population and to explore the dose-response relationship. This will directly address the current research gap and provide insights for future studies targeting NOD.

## 2. Methods

### 2.1 Study Population

We used data from participants in the National Health and Nutrition Examination Survey (NHANES), a well-established series of repeated cross-sectional surveys conducted in the United States. These surveys employ multistage probabilistic sampling strategies to select participants, including, where appropriate, oversampling of specific population segments [20]. The underlying protocol was approved by the research ethics review board of the National Center for Health Statistics (NCHS) [20]. The NHANES database is a publicly accessible resource, and all participants signed informed consent forms. The project underwent an ethical review, as detailed in the supplementary materials. For this study, we utilized NHANES data spanning from 1999 to 2018, selecting individuals who were taking statins, those without diabetes, and individuals with a confirmed diagnosis of diabetes and documented time of statin initiation, while excluding participants with a confirmed diagnosis of cancer. We identified the diagnosis of diabetes occurring after statin use as NOD.

### 2.2 Covariate Processing

We included indicators potentially related to NOD, encompassing participants’ demographic information such as age, sex, race, poverty income ratio (PIR), educational attainment (EDU), physical measurements (body mass index [BMI], waist circumference, blood pressure, physical activity, smoking status, alcohol consumption), medical history (hypertension, cardiovascular disease, heart failure, liver or kidney dysfunction), and laboratory parameters: triglyceride (TG), total cholesterol (TC), high-density lipoprotein cholesterol (HDL-C), low-density lipoprotein cholesterol (LDL-C), plasma glucose, hemoglobin A1c (HbA1c), estimated glomerular filtration rate (eGFR). Regarding CoQ10, we incorporated the following variables: CoQ10 (CoQ10 administration over the past 30 days), CoQ10 daily dosage (average daily intake of CoQ10 over the past 30 days, in mg), CoQ10 daily dosage per kilogram of body weight (average daily intake of CoQ10 per kilogram of body weight over the past 30 days, in mg/kg), CoQ10 daily dosage per body surface area (average daily intake of CoQ10 per body surface area over the past 30 days, in mg/m^2^). The instruments and methods used for the laboratory tests and the rules for calculating the indicators used in this document are described in the supplementary documents.

A total of 4394 collected samples were included in the study for analysis. As NHANES employs a probability-based sampling approach, intricate adjustments like sample weights, strata, and primary sampling units were used to handle the complex survey design, including oversampling, non-response, and post-stratification. All statistical analyses considered the NHANES database’s intricate sampling design. Non-normally distributed continuous variables are presented as median (Q25, Q75). The Wilcoxon rank-sum test was used to contrast two data sets while the Kruskal-Wallis test was used to compare multiple sets. Categorical variables are shown as absolute values (percentages). Pearson’s chi-squared test examines non-ordered variables, the Wilcoxon rank-sum test compares ordinal variables, and the Kruskal-Wallis test compares multiple groups of ordinal variables. Analyses were conducted using R version 4.3.0 (R Foundation for Statistical Computing, Vienna, Austria), with significance set at *p* < 0.05 (two-sided). Univariate and multivariate logistics regression analyses and restricted cubic spline (RCS) analysis were conducted, adjusting for confounders in two models. Model 1 included age, sex, race, poverty index, and educational attainment. Model 2 added adjustments for medical history factors (hypertension, CVD, heart failure) and variables (waist circumference, LDL-C, and age when began to take statin) in the entire study. These two models were based on established methodologies outlined in prior studies as well as the statistical analyses presented in this study.

## 3. Results

### 3.1 Characteristics of the Study Participants

**Fig. 1.**
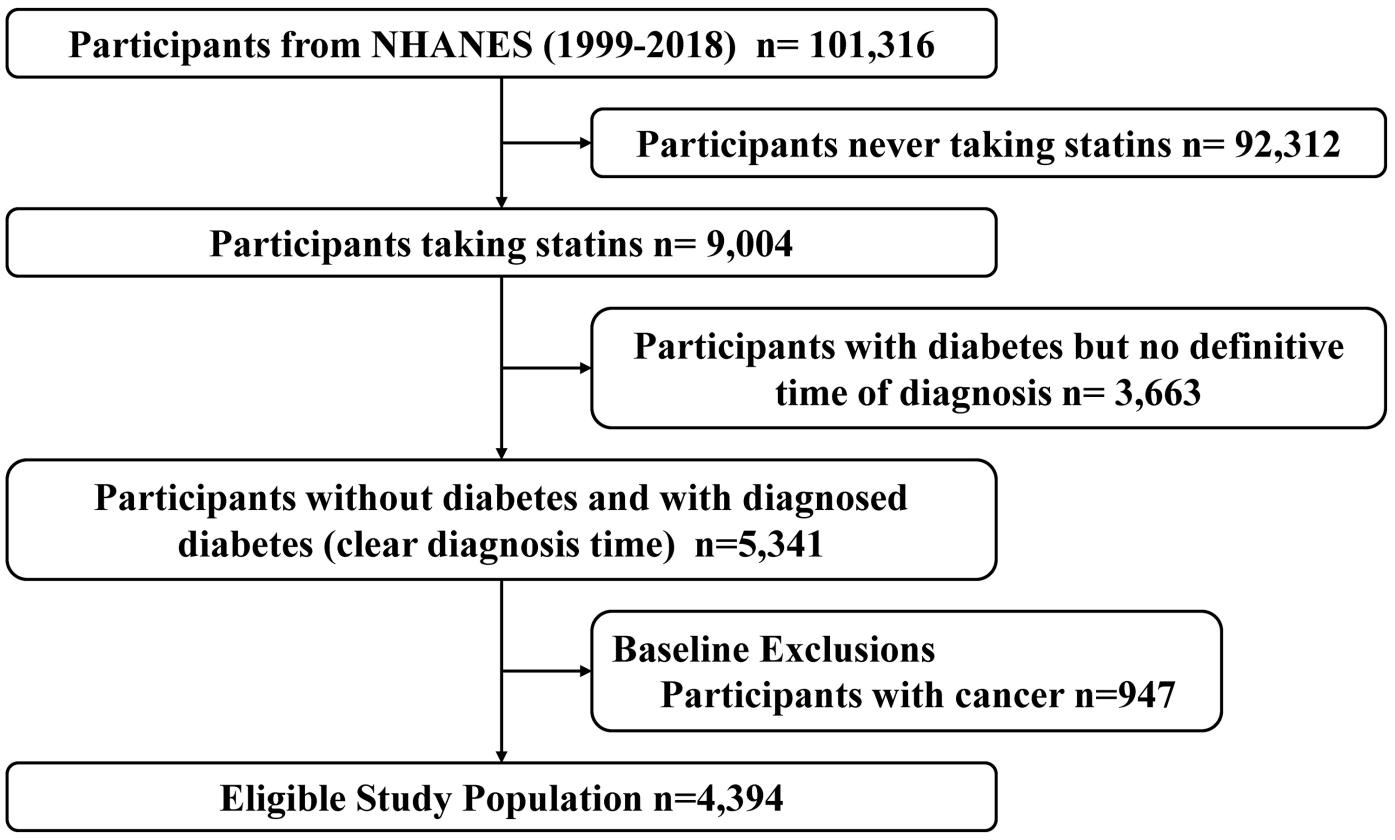
**Screening and selection process for study participants from NHANES**. NHANES, National Health and Nutrition Examination Survey.

This study ultimately included 4394 participants from NHANES 1999–2018 who met the inclusion and exclusion criteria. The median follow-up duration was 86.00 (46.00, 135.00) months. The specific screening process is outlined in Fig. 1.

The baseline characteristics of participants with and without NOD are summarized in Table 1. The mean age of the study population was 62.00 (54.00, 70.00) years, with females comprising 47% of the total sample. Among individuals on statin therapy, 276 (6.6%) developed NOD. This subgroup was characterized by older age (*p* = 0.010), a higher proportion of males (*p* = 0.007), and a greater prevalence of pre-existing conditions such as hypertension (*p* < 0.001), coronary heart disease (*p* < 0.001), and heart failure (*p* = 0.001). Additionally, they had larger waist circumference (*p* = 0.004), higher triglyceride levels (*p* = 0.042), and lower TC (*p* < 0.001), LDL-C (*p* < 0.001), and HDL-C levels (*p* < 0.001). Notably, those who developed NOD started statin therapy at a younger age (*p* < 0.001) and had a higher mortality rate associated with diabetes (*p* = 0.006). It is also significant that fewer participants in this subgroup took CoQ10 (*p* = 0.001), and the proportion taking a daily CoQ10 dose exceeding 30 mg was similarly lower (*p* = 0.001). However, there were no statistically significant differences in CoQ10 dosage between the two groups, whether assessed as daily intake or adjusted for body weight and body surface area.

**Table 1.** **Comparison of baseline characteristics for participants with and without NOD**.

Characteristic		Overall	Non-diabetes after statin	Diabetes after statin	*p* value
Individuals n (N)		4291 (15,582,680)	4015 (14,558,118)	276 (1,024,562)	-
Age (years)		62.00 (54.00, 70.00)	62.00 (54.00, 70.00)	64.00 (56.00, 72.00)	0.010
Sex (female)		2038 (47%)	1927 (48%)	111 (40%)	0.007
Race					0.233
	Non-hispanic white	1931 (45%)	1802 (45%)	129 (47%)	
	Non-hispanic black	954 (22%)	895 (22%)	59 (21%)	
	Mexican american	611 (14%)	584 (15%)	27 (9.8%)	
	Other/multiracial	422 (9.8%)	382 (9.5%)	40 (14%)	
	Other hispanic	373 (8.7%)	352 (8.8%)	21 (7.6%)	
PIR		3.07 (1.61, 5.00)	3.05 (1.60, 5.00)	3.43 (1.69, 5.00)	0.276
Educational attainment					0.345
	Below high school	700 (16%)	659 (16%)	41 (15%)	
	High school	1671 (39%)	1562 (39%)	109 (39%)	
	Above high school	1918 (45%)	1792 (45%)	126 (46%)	
Hypertension		3248 (77%)	3011 (76%)	237 (86%)	<0.001
CVD		705 (17%)	626 (16%)	79 (29%)	<0.001
Heart failure		471 (11%)	427 (11%)	44 (16%)	0.001
Smoking status					0.269
	Never smoker	2057 (48%)	1935 (48%)	122 (44%)	
	Former smoker	1598 (37%)	1472 (37%)	126 (46%)	
	Current smoker	636 (15%)	608 (15%)	28 (10%)	
Alcohol intake					<0.001
	Non-drinker	556 (19%)	532 (19%)	24 (13%)	
	Former drinker	222 (7.5%)	200 (7.2%)	22 (12%)	
	Drinker	2189 (74%)	2045 (74%)	144 (76%)	
Physical activity		78,240.00 (0.00, 283,518.15)	78,240.00 (0.00, 284,207.72)	76,047.74 (0.00, 273,798.43)	0.578
BMI (kg/m^2^)		29.78 (26.20, 34.50)	29.70 (26.14, 34.45)	31.07 (26.98, 35.10)	0.160
Waist circumference (cm)		105.20 (95.20, 116.00)	104.83 (95.00, 115.70)	109.20 (99.02, 118.17)	0.004
HbA1c (%)		5.80 (5.50, 6.30)	5.70 (5.40, 6.20)	6.70 (6.30, 7.10)	<0.001
TG (mmol/L)		1.39 (0.96, 1.98)	1.38 (0.96, 1.96)	1.59 (1.14, 2.07)	0.042
TC (mmol/L)		4.55 (3.93, 5.22)	4.60 (3.98, 5.22)	3.97 (3.59, 4.94)	<0.001
LDL-C (mmol/L)		2.48 (1.97, 3.00)	2.51 (1.99, 3.00)	2.07 (1.63, 2.54)	<0.001
HDL-C (mmol/L)		1.32 (1.09, 1.58)	1.32 (1.09, 1.60)	1.16 (1.06, 1.29)	<0.001
eGFR (mL/min)		172.90 (141.00, 202.82)	172.96 (141.29, 202.96)	168.35 (135.37, 194.61)	0.228
Age when first taking statin		58.00 (50.00, 66.00)	58.00 (50.00, 66.00)	53.00 (49.00, 61.00)	<0.001
CoQ10		127 (3.0%)	122 (3.0%)	5 (1.8%)	0.001
CoQ10 daily dosage (mg)		50.00 (21.00, 100.00)	50.00 (21.72, 100.00)	45.19 (7.45, 101.75)	0.604
CoQ10 daily dosage per kilogram of body weight (mg/kg)		17.00 (6.48, 41.70)	16.88 (6.50, 41.63)	13.06 (2.15, 28.95)	0.889
CoQ10 daily dosage per body surface area (mg/m^2^)		723.02 (289.45, 1613.47)	721.79 (313.34, 1613.12)	588.69 (98.49, 1301.59)	>0.978
All-cause mortality		1089 (25%)	1014 (25%)	75 (27%)	0.766
Cardiovascular mortality		342 (8.0%)	312 (7.8%)	30 (11%)	0.202
Cancer-related mortality		166 (3.9%)	157 (3.9%)	9 (3.3%)	>0.904
Diabetes-related mortality		228 (5.3%)	203 (5.1%)	25 (9.1%)	0.006
Survival length (months)		86.00 (46.00, 135.00)	89.00 (47.00, 138.00)	62.00 (36.00, 100.00)	<0.001

BMI, body mass index; CoQ10, coenzyme Q10; CVD, cardiovascular disease; eGFR, estimated glomerular filtration rate; HbA1c, hemoglobin A1c; HDL-C, high-density lipoprotein cholesterol; LDL-C, low-density lipoprotein cholesterol; PIR, poverty income ratio; TC, total cholesterol; TG, triglycerides; NOD, new-onset diabetes. n represents the number of participants in the study sample, while N represents the weighted number of participants based on NHANES sampling principles. Continuous variables are presented as median (Q25, Q75). Categorical variables are presented as numbers (percentages).

Table 2 presents a comparison of baseline characteristics between individuals who took CoQ10 and those who did not. In this study, 127 participants (3.8%) reported CoQ10 supplementation. This subgroup was distinguished by significantly higher socioeconomic status (*p* < 0.001), a greater proportion with higher educational attainment (*p* < 0.001), a lower prevalence of diabetes (*p* = 0.003), reduced HbA1c levels (*p* = 0.032), a later age of diabetes onset (*p* = 0.010), and a lower incidence of NOD following statin therapy (*p* = 0.001). For a more detailed comparison of the two cohorts, refer to **Supplementary Table 1**.

**Table 2.** **Comparison of baseline characteristics between participants taking CoQ10 and those not taking CoQ10**.

Characteristic		Non-CoQ10	Taking CoQ10	*p* value
Individuals n (N)		4164 (14,990,114)	127 (592,567)	-
Age (years)		62.00 (54.00, 70.00)	65.41 (57.00, 71.00)	0.075
Sex (female)		1972 (47%)	66 (52%)	0.2
PIR		3.04 (1.58, 5.00)	4.30 (2.50, 5.00)	<0.001
Educational attainment				<0.001
	Below high school	695 (17%)	5 (3.9%)	
	High school	1627 (39%)	44 (35%)	
	Above high school	1840 (44%)	78 (61%)	
Diabetes		2404 (58%)	57 (45%)	0.003
HbA1c (%)		5.80 (5.50, 6.30)	5.70 (5.43, 5.90)	0.032
Age when first diagnosed with diabetes		50.00 (40.00, 59.00)	55.01 (50.00, 60.00)	0.010
New onset diabetes after statin use		271 (6.5%)	5 (3.9%)	0.001

n represents the number of participants in the study sample, while N represents the weighted number of participants based on NHANES sampling principles. Continuous variables are presented as median (Q25, Q75). Categorical variables are presented as numbers (percentages).

### 3.2 Association of CoQ10 with New-onset Diabetes

We conducted one-way logistic regression to identify the factors associated with NOD. The statistically significant indicators, along with their corresponding odds ratios (OR) and 95% confidence intervals (CI) are presented in Table 3. The complete results of the one-way logistic regression can be found in **Supplementary Table 2**. Notably, CoQ10 use (*p* = 0.003) was significantly associated with NOD. However, the specific dosage of CoQ10 did not demonstrate a linear correlation with the incidence of NOD.

**Table 3.** **Results of univariate logistic regression identifying factors associated with NOD**.

Characteristic		OR	95% CI	*p* value
Age (years)		1.019	1.006, 1.031	0.003
Sex (male)		1.657	1.147, 2.394	0.007
Race				
	Mexican american	—	—	
	Other hispanic	1.899	0.837, 4.310	0.124
	Non-hispanic white	2.033	1.199, 3.449	0.009
	Non-hispanic black	1.832	1.050, 3.196	0.033
	Other/multiracial	2.537	1.459, 4.412	0.001
PIR		1.053	0.948, 1.170	0.331
Educational attainment				
	Below high school	—	—	
	High school	1.150	0.730, 1.810	0.545
	Above high school	1.287	0.829, 1.998	0.259
Hypertension		2.842	1.554, 5.197	<0.001
CVD		2.344	1.443, 3.809	<0.001
Heart failure		2.124	1.343, 3.361	0.001
Waist circumference (cm)		1.015	1.006, 1.025	0.002
TG (mmol/L)		1.054	0.985, 1.128	0.124
TC (mmol/L)		0.621	0.502, 0.767	<0.001
LDL-C (mmol/L)		0.591	0.433, 0.807	0.001
HDL-C (mmol/L)		0.179	0.084, 0.383	<0.001
eGFR (mL/min)		0.998	0.995, 1.001	0.207
Age when first taking statin		0.971	0.959, 0.984	<0.001
CoQ10		0.323	0.157, 0.668	0.003
CoQ10 daily dosage (mg)		0.999	0.994, 1.004	0.720
CoQ10 daily dosage per kilogram of body weight (mg/kg)		0.991	0.973, 1.010	0.341
CoQ10 daily dosage per body surface area (mg/m^2^)		1.000	0.999, 1.000	0.456

**Fig. 2.**
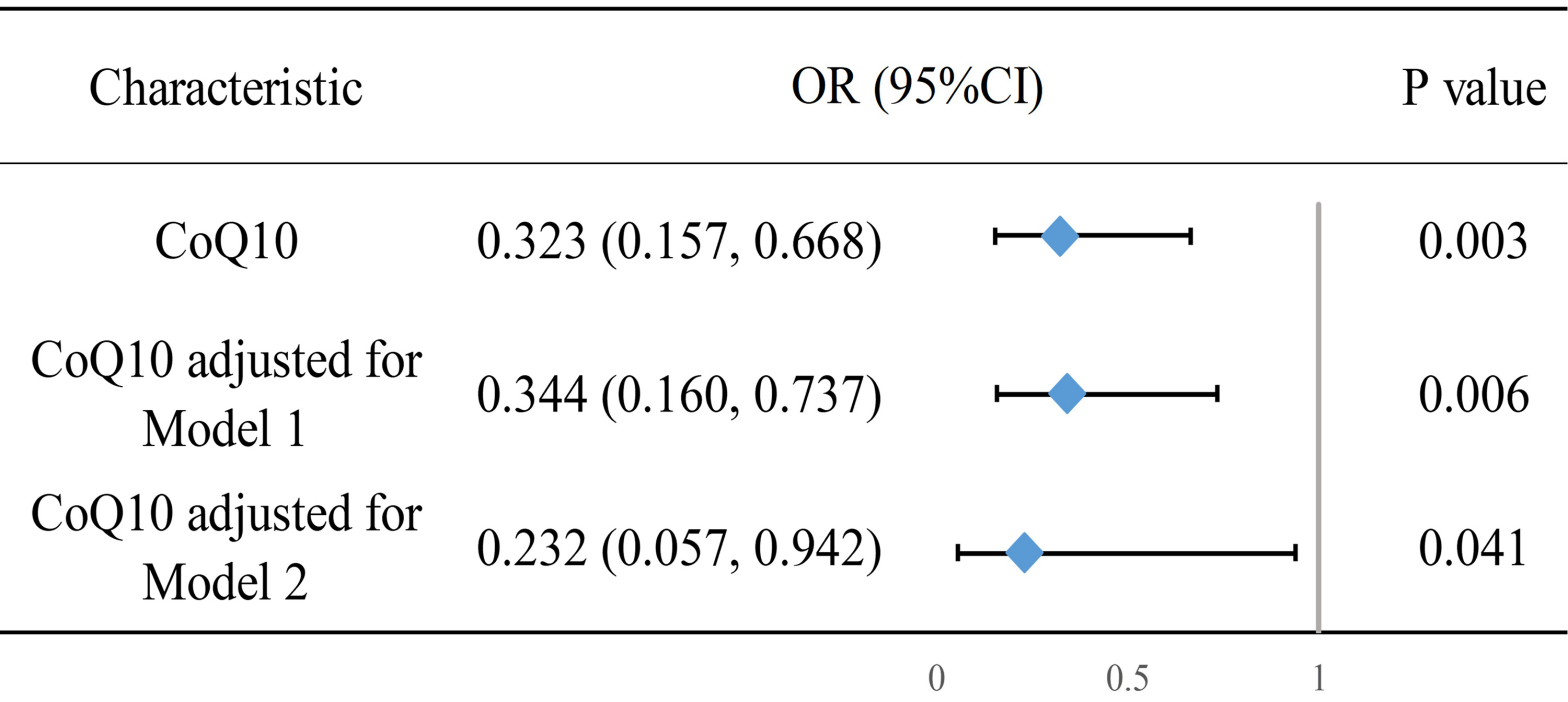
**Forest plot of multifactorial logistic regression results of CoQ10 and NOD**. Model 1 adjusts for age, sex, race, PIR, and educational attainment. Model 2 further adjusts for hypertension, CVD, heart failure, waist circumference, LDL-C, and the age at which statin therapy was initiated; OR, odds ratio.

Based on the aforementioned results, we developed two models to correct for confounding factors and conducted multifactor logistic regression. The specific regression values are shown in **Supplementary Table 3**. To illustrate the role of CoQ10 more clearly, we included it in a forest plot (Fig. 2). A post hoc power analysis of the weighted statistics yielded a power value of 1.0. We also employed the variance inflation factor to assess multicollinearity among the covariates in the logistic regression, with the results detailed in **Supplementary Table 4**.

### 3.3 Association of CoQ10 Daily Dosage with New-onset Diabetes

**Fig. 3.**
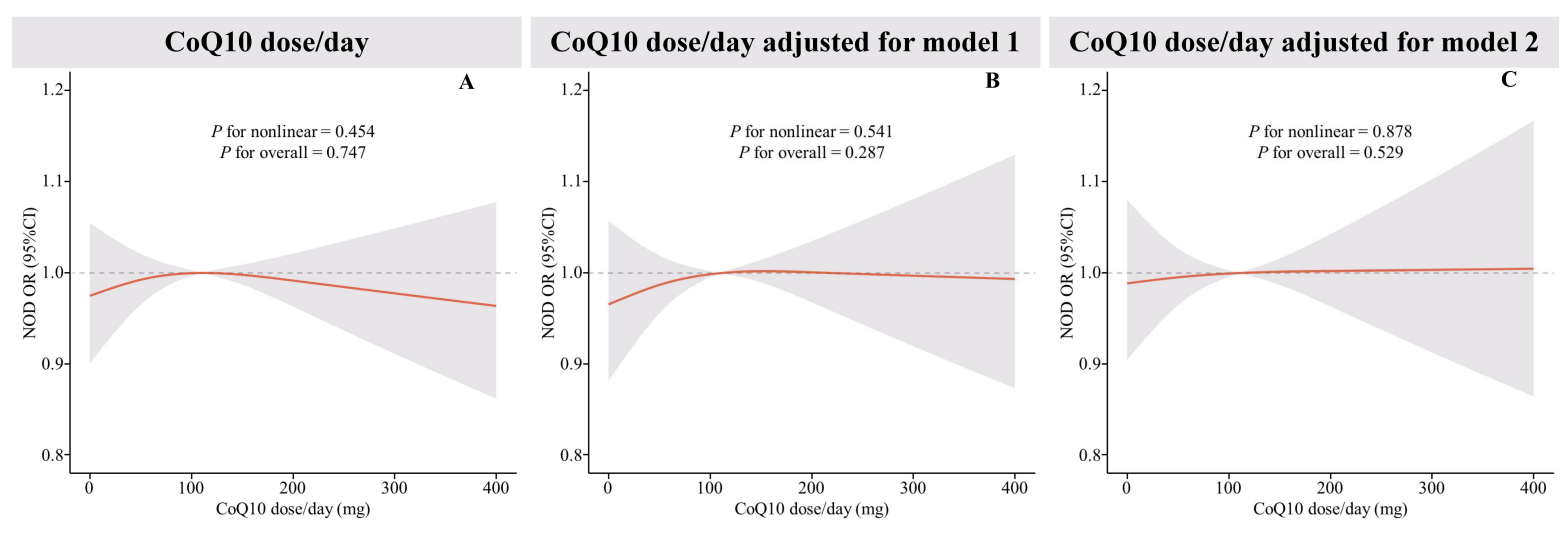
**Restricted cubic spline (RCS) plot of the relationship between CoQ10 daily dose and new-onset diabetes**. (A) RCS curves of the correlation between CoQ10 daily dose and new-onset diabetes were shown, (B) RCS curves of the correlation between CoQ10 daily dose and new-onset diabetes under Model 1 adjustment, and (C) RCS curves of the correlation between CoQ10 daily dose and new-onset diabetes under Model 2 adjustment. The red lines represent odds ratios and shading indicates 95% confidence intervals. New onset diabetes was derived via logistics regression using RCS, with adjustments made in model 1 and model 2. Model 1 accounts for age, sex, race, PIR, and educational attainment. Model 2 further adjusts for hypertension, CVD, heart failure, waist circumference, LDL-C, and the age at which statin therapy was initiated.

The RCS analysis (Fig. 3) did not find a nonlinear correlation between CoQ10 daily dose and NOD. This lack of correlation persisted even after multiple modeling adjustments. Further investigated the relationship between daily CoQ10 dose per unit of body weight and per unit of body surface area with NOD was conducted using RCS (**Supplementary Fig. 1**). These analyses also demonstrated no statistically significant nonlinear correlation (*p* > 0.05).

### 3.4 Sensitivity Testing

In our further analysis, we explored the interaction between CoQ10 and various factors, including age, BMI, CVD, waist circumference, hypertension, heart failure, and LDL-C. The results are presented in **Supplementary Table 5**. Notably, the data suggested an interaction between CoQ10 and the incidence of NOD, specifically with heart failure. However, due to the limited number of participants, subgroup analyses were not feasible. To overcome this limitation, we implemented propensity score matching based on demographic characteristics, including age, sex, race, PIR, and educational attainment. After matching, CoQ10’s impact on NOD was reevaluated using multifactorial logistic regression. A 1:1 matching for CoQ10 users versus non-users yielded an OR of 0.061 (95% CI: 0.006–0.336, *p* = 0.006), indicating a significant protective effect of CoQ10. A similar protective effect was observed in the 1:2 matching, which yielded an OR of 0.197 (95% CI: 0.051–0.610, *p* = 0.010). Both sensitivity test results were consistent with the conclusions of this study.

## 4. Discussion

This study retrospectively included a total of 4394 patients from the NHANES database spanning the years 1999 to 2018, with a median follow-up duration of 86.00 (46.00, 135.00) months. The key finding is that among patients taking statin medications, the simultaneous co-administration of CoQ10 is associated with a reduction in NOD. This correlation remained significant even after adjusting for various confounding factors (*p* < 0.05). Further analyses confirmed that the observed correlation and protective effect is independent of the dosage of CoQ10 supplementation.

While statin therapies are widely utilized in clinical practice, multiple studies have confirmed their association with NOD risk [3, 4, 5, 21, 22, 23], which may be related to CoQ10 deficiency. Research has demonstrated that statin drugs can decrease CoQ10 levels in the blood by 16% to 54% [24]. Two possible mechanisms contribute to the statin-induced reduction in CoQ10. Firstly, statin treatment inhibits the production of mevalonate, an intermediate in CoQ10 synthesis. Secondly, statin drugs lower LDL-C levels, potentially impacting CoQ10 transport. External supplementation of CoQ10 has been shown to elevate the levels of CoQ10 in the blood of patients undergoing statin therapy [24, 25]. Additionally, CoQ10 deficiencies can lead to impaired mitochondrial function and reduced antioxidant capacity, further decreasing insulin secretion and sensitivity, which may contribute to NOD development [25, 26]. Exogenous supplementation with CoQ10 may ameliorate this process [16]. In addition, studies have confirmed that supplementation with CoQ10 improves mitochondrial metabolism, which plays a crucial role in the pathogenesis of diabetes mellitus [19, 27].

Therefore, we hypothesized that exogenous supplementation with CoQ10 might help reduce the occurrence of NOD in those taking statins [28]. In previous animal experiments, Lorza-Gil *et al*. [16] found that pravastatin-treated hypercholesterolemic LDL receptor knockout mice exhibited decreased insulin secretion, increased islet cell death, and increased oxidative stress. Initiating dietary supplementation of CoQ10 in these mice reversed fasting hyperglycemia, improved glucose tolerance (by 20%), enhanced insulin sensitivity (by >2-fold), and fully restored the damage caused by pravastatin-impaired pancreatic glucose-stimulated insulin secretion by 40% [16]. Subsequent *in vitro* experiments also confirmed that co-treatment of insulin-secreting INS1E cells with CoQ10 protected the cells from pravastatin-induced cell death and oxidative stress. Simvastatin and atorvastatin were even more effective (10- to 15-fold) in inducing dose-dependent INS1E cell death, which was also mitigated by CoQ10 co-treatment [16]. This study demonstrated that statins impair β-cell redox homeostasis, function, and viability while CoQ10 supplementation protects against the deleterious effects of statins on pancreatic endocrine secretion [16]. However, limited clinical studies have been conducted on the effect of exogenous CoQ10 supplementation on statin-induced NOD. The 2019 LIFESTAT study, which included people using simvastatin for primary prevention, found that the insulin secretory capacity was unaltered by supplementation with CoQ10 (400 mg/day). Although insulin sensitivity remained unchanged, hepatic insulin sensitivity was elevated [29]. Besides, several studies have confirmed that CoQ10 supplementation can reduce glucose and HbA1c levels [25, 26, 30, 31, 32].

Given the limited number of studies investigating CoQ10 supplementation in the context of NOD among statin-using populations, we must refer to related studies for insights into CoQ10 dosage. In statin-related studies, Fedacko *et al*. [33] found significant improvement in statin-related myopathy with a CoQ10 dosage of 200 mg/day over three months. Similarly, another study demonstrated that CoQ10 supplementation (240 mg/d for 22 months) markedly reduced the incidence of statin-related fatigue (from 84% to 16%) and myalgia (from 58% to 6%) [34]. Additionally, several studies have shown that CoQ10 supplementation at a dose of 150 mg/day or 200 mg/day for three months significantly improves fasting blood glucose and HbA1c levels [30, 31, 32]. This study found no statistically significant correlation between CoQ10 dosage and the incidence of NOD. We attribute this finding to several factors: (1) The retrospective design of this study posed several limitations, particularly with the small number of participants taking CoQ10 and the broad range of CoQ10 dosages, which may have obscured statistical differences. (2) The study relied on the average CoQ10 dose taken in the past month, which may not accurately reflect the total CoQ10 exposure over the entire period of statin therapy. We recommend future well-designed randomized controlled trials to explore the dose-response relationship between CoQ10 and NOD in statin users. Identifying the optimal dosage would provide a practical and effective means to prevent NOD in this population. A key strength of this study is that this is the first to leverage the NHANES database to investigate the association between exogenous CoQ10 supplementation and NOD in a statin-using population. The NHANES database’s robust sampling methodology, combined with the appropriate use of statistical weighting, allows the findings of this study to extend beyond the sampled cohort, and to be generalized to the entire U.S. population using statins. This broad generalizability enhances the sensitivity and reliability of the study’s conclusions, making its findings particularly valuable for public health applications.

Despite the aforementioned strengths, this study does have notable limitation. As a retrospective study, it is inherently constrained by the reliance on observational data, making it difficult to establish causality between CoQ10 supplementation and NOD—we can only infer a correlation. Furthermore, the small sample size of participants taking CoQ10, compared to those who were not, may have increased the margin of error in the statistical analysis of CoQ10’s effects. Additionally, several continuous variables in the analysis do not follow a normal distribution. This deviation necessitated the use of non-parametric tests, reducing the statistical power of some comparisons. Another limitation is the lack of data on the total duration of CoQ10 administration and serum CoQ10 concentrations, which could have provided more precise insights into CoQ10’s role. These limitations are common in retrospective analyses, and we hope that well-designed randomized controlled trials in the future will further explore the qualitative and quantitative relationships between CoQ10 supplementation and NOD risk in statin users.

The opportunity presented by this study is its suggestion of a potentially effective approach for preventing NOD in individuals who require statin therapy, but are concerned about the risk of developing NOD. This could enable more patients to benefit from the cardiovascular protective effects of statins without fear of adverse metabolic effects. Additionally, this research sets a foundation for future randomized controlled trials to further investigate the dose-response relationship between CoQ10 supplementation and NOD, providing a clear direction for subsequent studies.

## 5. Conclusions

In individuals taking statins, CoQ10 supplementation is associated with a reduced risk of developing NOD. This relationship remains statistically significant even after adjusting for confounding factors (*p* < 0.05). Interestingly, the effect of CoQ10 on reducing NOD risk does not appear to be influenced by the dosage of CoQ10.

## Data Availability

Data described in the manuscript comes from the official NHANES website at https://wwwn.cdc.gov/nchs/nhanes/continuousnhanes/default.aspx. If needed, the corresponding author can provide the relevant R code upon request. This study is an observational retrospective study, and according to the Clinical Trial Registration Statement from the International Committee of Medical Journal Editors (ICMJE), registration is not required for purely observational studies.

## Abbreviations

ATP, adenosine triphosphate; BMI, body mass index; CoQ10, coenzyme Q10; CVD, cardiovascular disease; eGFR, estimated glomerular filtration rate; HbA1c, hemoglobin A1c; HDL-C, high-density lipoprotein cholesterol; LDL-C, low-density lipoprotein cholesterol; NCHS, National Center for Health Statistics; NHANES, National Health and Nutrition Examination Survey; NOD, new-onset diabetes; PIR, poverty income ratio; RCS, restricted cubic spline; TG, triglyceride; TC, total cholesterol.

## Supplementary Material

Supplementary material associated with this article can be found, in the online version, at https://doi.org/10.31083/RCM26437.
