# Supplementary material for: Diabetes Risks of Statin Therapy—Coenzyme Q10 May Help
Source: Rev Cardiovasc Med. 2025 Feb 19;26(2):26437. doi: 10.31083/RCM26437 (PMC11868890; doi:10.31083/RCM26437)
Supplement: Supplementary file 1 [file 2153-8174-26-2-26437-s1.docx]

**Covariate processing**

In NHANES, the data collection process consists of three main phases. The first phase involves household interviews where information on various factors such as age, gender, race, economic status, education level, diet, and health history are collected through questionnaires. The second phase takes place at a mobile screening center and focuses on collecting physical examination data, including measurements of height, weight, BMI, etc. The third phase involves telephone follow-up, primarily for dietary purposes. Body mass index (BMI) was calculated as weight in kilograms divided by the square of height in meters. The poverty impact ratio (PIR) represents the family income-to-poverty ratio. Smoking status included never smoker (≤100 cigarettes lifetime), former smoker (>100 cigarettes lifetime, currently quit), and current smoker. Alcohol consumption categories were non-drinkers, former drinkers, and drinkers. Physical activity, measured in metabolic equivalent of task (MET), indicated average weekly calories burned. Diabetes was defined by self-report, insulin use, fasting glucose>7mmol/L, or HbA1c≥6.5%. Hypertension included self-reported history, SBP≥140mmHg, or DBP≥90mmHg. History of CVD, heart failure, and stroke was self-reported. Impaired renal function was self-reported or eGFR >90ml/min/1.73m². The eGFR is calculated according to the classic MDRD formula, the formula being: eGFR = 170 × Scr ^ -0. 999 × Age ^ -0.176 × BUN ^ -0.170 × ALB ^ 0.318 × 0.762 (females) × l.180 (Afro-descendants). The use of statins, fibrate, and niacin was based on medication inventory.

The laboratory tests referenced in this article include the following key indicators: triglycerides, total cholesterol, high-density lipoprotein cholesterol (HDL-C), low-density lipoprotein cholesterol (LDL-C), plasma glucose, glycohemoglobin (HbA1c), creatinine, urea nitrogen, and albumin. The detailed methods and instruments used for these tests are summarized in the table below, showcasing the specific analytical techniques and equipment involved in measuring these clinical parameters to ensure accurate and standardized results.

| Characteristic | Method | Equipment | Reagents | Website |
| --- | --- | --- | --- | --- |
| TG | Cobas 6000 Chemistry Analyzer | Roche/Hitachi Cobas 6000 Analyzer | Cell Wash Solution II/Acid Wash, Cell Wash Solution I/NaOH-D, ECOTergent/Hitergent/Eco-D | extension://bfdogplmndidlpjfhoijckpakkdjkkil/pdf/viewer.html?file=https%3A%2F%2Fwwwn.cdc.gov%2Fnchs%2Fdata%2Fnhanes%2F2017-2018%2Flabmethods%2FTRIGLY-J-MET-508.pdf |
| TC | Roche Cobas 6000 | Roche/Hitachi Cobas 6000 Analyze | Cobas 6000 system reagents | extension://bfdogplmndidlpjfhoijckpakkdjkkil/pdf/viewer.html?file=https%3A%2F%2Fwwwn.cdc.gov%2Fnchs%2Fdata%2Fnhanes%2F2017-2018%2Flabmethods%2FTCHOL-J-MET-508.pdf |
| HDL-C | Cobas 6000 Chemistry Analyzer | Roche/Hitachi Cobas 6000 Analyzer | Cobas 6000 system reagents | extension://bfdogplmndidlpjfhoijckpakkdjkkil/pdf/viewer.html?file=https%3A%2F%2Fwwwn.cdc.gov%2Fnchs%2Fdata%2Fnhanes%2F2017-2018%2Flabmethods%2FHDL-J-MET-508.pdf |
| LDL-C | LDL-C is calculated from directly measured values of total cholesterol (LBXTC), triglycerides (LBXTR), and HDL-C (LBDHDD). | | | https://wwwn.cdc.gov/Nchs/Nhanes/2017-2018/TRIGLY_J.htm |
| plasma glucose | Roche/Hitachi Cobas C Chemistry Analyzer – C311 | For cobas c 311 analyzers: GLUC3. | REF 04404483, CONTENT Glucose HK (GLUC3) | extension://bfdogplmndidlpjfhoijckpakkdjkkil/pdf/viewer.html?file=https%3A%2F%2Fwwwn.cdc.gov%2Fnchs%2Fdata%2Fnhanes%2F2017-2018%2Flabmethods%2FGLU-J-MET-508.pdf |
| HbA1c | Trinity Biotech Boronate Affinity HPLC | Trinity Biotech Premier Hb9210 Automated HPLC System | Trinity | extension://bfdogplmndidlpjfhoijckpakkdjkkil/pdf/viewer.html?file=https%3A%2F%2Fwwwn.cdc.gov%2Fnchs%2Fdata%2Fnhanes%2F2017-2018%2Flabmethods%2FGHB-J-Premier-508.pdf |
| Scr | Roche Cobas 6000 (c501 module) | Roche Cat. No. 03263991190 | CREP2 reagent kit (250 tests) | extension://bfdogplmndidlpjfhoijckpakkdjkkil/pdf/viewer.html?file=https%3A%2F%2Fwwwn.cdc.gov%2Fnchs%2Fdata%2Fnhanes%2F2017-2018%2Flabmethods%2FBIOPRO-J-MET-Creatinine-508.pdf |
| Bun | Roche Cobas 6000 (c501 module) | Roche Cat. No. 04460715190 | UREAL reagent kit (500 tests) | extension://bfdogplmndidlpjfhoijckpakkdjkkil/pdf/viewer.html?file=https%3A%2F%2Fwwwn.cdc.gov%2Fnchs%2Fdata%2Fnhanes%2F2017-2018%2Flabmethods%2FBIOPRO-J-MET-BUN-508.pdf |
| ALB | Roche Cobas 6000 (c501 module) | Roche product #05599261190 | ALBP reagent kit (225 tests) | extension://bfdogplmndidlpjfhoijckpakkdjkkil/pdf/viewer.html?file=https%3A%2F%2Fwwwn.cdc.gov%2Fnchs%2Fdata%2Fnhanes%2F2017-2018%2Flabmethods%2FBIOPRO-J-MET-Albumin-508.pdf |

**NCHS Ethics Review Board (ERB) Approval**

The NHANES database has received NCHS ERB Approval, which is available on the official website at https://www.cdc.gov/nchs/nhanes/irba98.htm#print, and excerpted information is below:


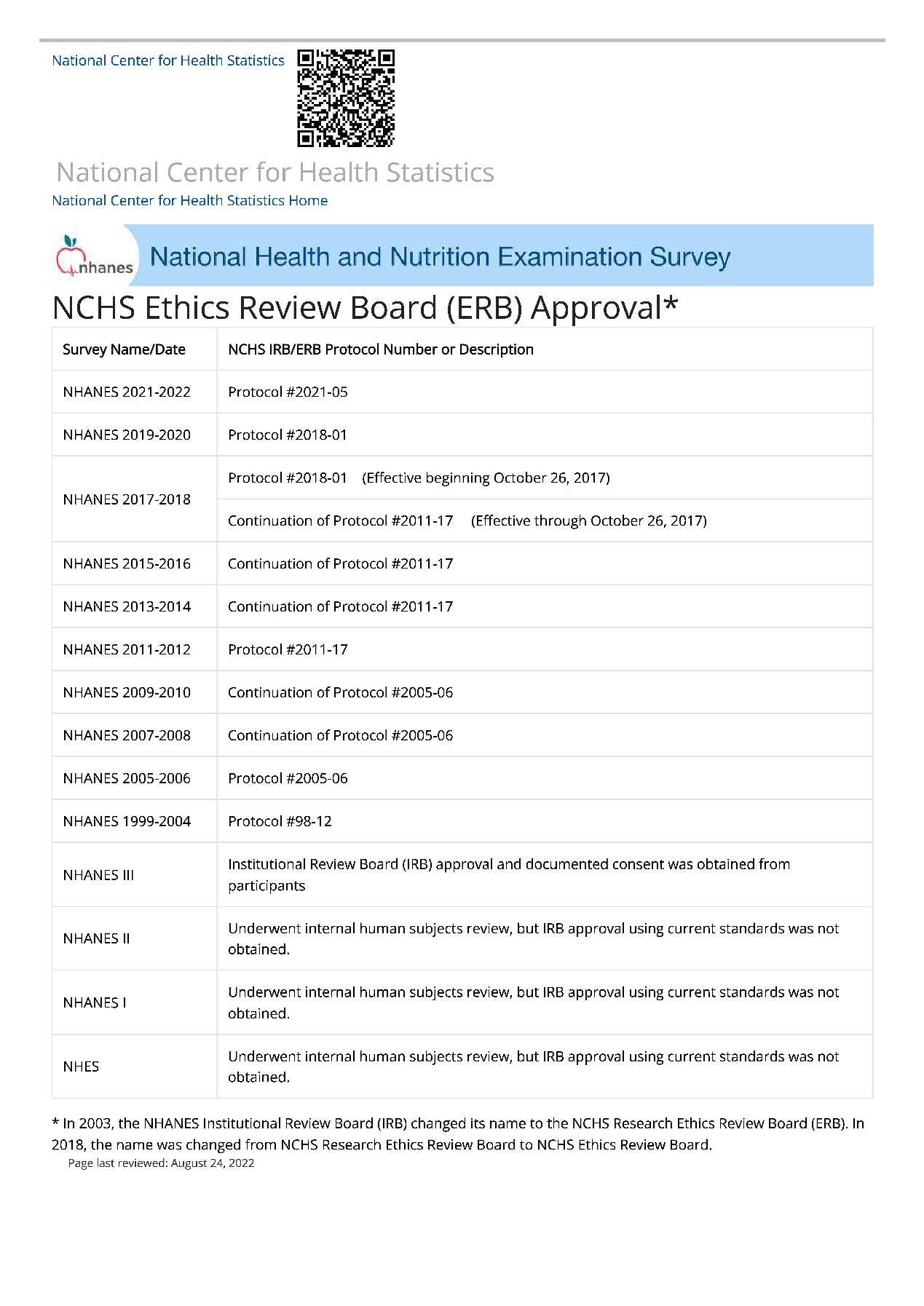


**Supplementary Table 1** Comparison of baseline characteristics between CoQ10 users and non-users

| **Characteristic** | **Non-CoQ10** | **Taking CoQ10** | ***P* value** |
| --- | --- | --- | --- |
| Individuals n (N) | 4164 (14,990,114) | 127 (592,567) | - |
| Age (years) | 62.00 (54.00, 70.00) | 65.41 (57.00, 71.00) | 0.075 |
| Gender (female) | 1,972 (47%) | 66 (52%) | 0.2 |
| Race |  |  | 0.003 |
| Non-Hispanic White | 1,855 (45%) | 76 (60%) |  |
| Non-Hispanic Black | 934 (22%) | 20 (16%) |  |
| Mexican American | 602 (14%) | 9 (7.1%) |  |
| Other/multiracial | 405 (9.7%) | 17 (13%) |  |
| Other Hispanic | 368 (8.8%) | 5 (3.9%) |  |
| PIR | 3.04 (1.58, 5.00) | 4.30 (2.50, 5.00) | <0.001 |
| Educational attainment |  |  | <0.001 |
| Below high school | 695 (17%) | 5 (3.9%) |  |
| High school | 1,627 (39%) | 44 (35%) |  |
| Above high school | 1,840 (44%) | 78 (61%) |  |
| Hypertension | 3,156 (77%) | 92 (74%) | 0.9 |
| Cardiovascular disease | 678 (16%) | 27 (21%) | >0.9 |
| Heart failure | 464 (11%) | 7 (5.5%) | 0.3 |
| Chronic bronchitis | 373 (9.0%) | 5 (4.0%) | 0.10 |
| Liver condition | 215 (5.2%) | 5 (4.0%) | 0.077 |
| Stroke | 458 (11%) | 5 (3.9%) | 0.2 |
| A weak or failing kidney | 460 (12%) | 8 (6.5%) | 0.040 |
| Smoking status |  |  | 0.3 |
| Never smoker | 1,987 (48%) | 70 (55%) |  |
| Former smoker | 1,551 (37%) | 47 (37%) |  |
| Current smoker | 626 (15%) | 10 (7.9%) |  |
| Alcohol intake |  |  | 0.5 |
| Non-drinker | 545 (19%) | 11 (13%) |  |
| Former drinker | 213 (7.4%) | 9 (11%) |  |
| Drinker | 2,124 (74%) | 65 (76%) |  |
| Physical activity | 77,112.52 (0.00, 280,357.43) | 101,353.37 (11,992.04, 369,214.35) | 0.10 |
| BMI (kg/m^2^) | 29.79 (26.20, 34.50) | 29.32 (25.94, 35.22) | >0.9 |
| Waist circumference (cm) | 105.20 (95.30, 116.00) | 103.60 (92.89, 114.68) | 0.5 |
| TG (mmol/L) | 1.39 (0.97, 1.98) | 1.26 (0.89, 2.05) | 0.7 |
| TC (mmol/L) | 4.55 (3.93, 5.22) | 4.65 (4.16, 5.28) | 0.062 |
| LDL-C (mmol/L) | 2.48 (1.97, 3.00) | 2.53 (2.25, 2.96) | 0.2 |
| HDL-C (mmol/L) | 1.32 (1.09, 1.58) | 1.34 (1.18, 1.71) | 0.052 |
| eGFR (ml/min) | 173.02 (141.02, 203.48) | 166.65 (138.60, 190.90) | 0.2 |
| Age when first taking statin | 57.00 (50.00, 66.00) | 59.00 (53.00, 66.00) | 0.12 |
| Diabetes | 2,404 (58%) | 57 (45%) | 0.003 |
| Glucose (mmol/L) | 5.91 (5.42, 6.75) | 6.05 (5.44, 6.37) | >0.9 |
| HbA1c (%) | 5.80 (5.50, 6.30) | 5.70 (5.43, 5.90) | 0.032 |
| Age when first diagnosed with diabetes | 50.00 (40.00, 59.00) | 55.01 (50.00, 60.00) | 0.010 |
| New onset diabetes after statin use | 271 (6.5%) | 5 (3.9%) | 0.001 |
| All-cause mortality | 1,066 (26%) | 23 (18%) | 0.3 |
| Cardiovascular mortality | 337 (8.1%) | 5 (3.9%) | 0.2 |
| Cancer-related mortality | 161 (3.9%) | 5 (3.9%) | >0.9 |
| Diabetes-related mortality | 225 (5.4%) | 3 (2.4%) | 0.8 |
| Survival length (months) | 87.00 (47.00, 135.00) | 80.40 (37.00, 144.98) | 0.5 |

BMI: body mass index, CI: Confidence Interval, CoQ10: coenzyme Q10, eGFR: estimated glomerular filtration rate, HbA1c: glycohemoglobin, HDL-C: high-density lipoprotein cholesterol, LDL-C: low-density lipoprotein cholesterol, OR: Odds Ratio, PIR: poverty income ratio, TC: total cholesterol, TG: triglycerides. n represents the number of participants in the study sample, while N represents the weighted number of participants based on NHANES sampling principles. Continuous variables are presented as median (Q25, Q75). Categorical variables are presented as numbers (percentages).

**Supplementary Table 2** Univariate logistic regression results for factors associated with new-onset diabetes.

| **Characteristic** | **OR** | **95% CI** | ***P* value** |
| --- | --- | --- | --- |
| Age (years) | 1.019 | 1.006, 1.031 | 0.003 |
| Gender (male) | 1.657 | 1.147, 2.394 | 0.007 |
| Race |  |  |  |
| Mexican American | — | — |  |
| Other Hispanic | 1.899 | 0.837, 4.310 | 0.124 |
| Non-Hispanic White | 2.033 | 1.199, 3.449 | 0.009 |
| Non-Hispanic Black | 1.832 | 1.050, 3.196 | 0.033 |
| Other/multiracial | 2.537 | 1.459, 4.412 | 0.001 |
| PIR | 1.053 | 0.948, 1.170 | 0.331 |
| Educational attainment |  |  |  |
| Below high school | — | — |  |
| High school | 1.150 | 0.730, 1.810 | 0.545 |
| Above high school | 1.287 | 0.829, 1.998 | 0.259 |
| Hypertension | 2.842 | 1.554, 5.197 | <0.001 |
| Cardiovascular disease | 2.344 | 1.443, 3.809 | <0.001 |
| Heart failure | 2.124 | 1.343, 3.361 | 0.001 |
| Smoking status |  |  |  |
| Never smoker | — | — |  |
| Former smoker | 1.483 | 1.053, 2.088 | 0.024 |
| Current smoker | 1.009 | 0.537, 1.894 | 0.978 |
| Alcohol intake |  |  |  |
| Non-drinker | — | — |  |
| Former drinker | 2.972 | 1.398, 6.317 | 0.005 |
| Drinker | 2.857 | 1.693, 4.824 | <0.001 |
| Physical activity | 1.000 | 1.000, 1.000 | 0.156 |
| BMI (kg/m2) | 1.019 | 0.995, 1.044 | 0.117 |
| Waist circumference (cm) | 1.015 | 1.006, 1.025 | 0.002 |
| SBP (mmHg) | 1.008 | 1.001, 1.014 | 0.022 |
| DBP (mmHg) | 1.006 | 0.992, 1.020 | 0.402 |
| Glucose (mmol/L) | 1.192 | 1.133, 1.254 | <0.001 |
| HbA1c (%) | 1.413 | 1.291, 1.547 | <0.001 |
| TG (mmol/L) | 1.054 | 0.985, 1.128 | 0.124 |
| TC (mmol/L) | 0.621 | 0.502, 0.767 | <0.001 |
| LDL-C (mmol/L) | 0.591 | 0.433, 0.807 | 0.001 |
| HDL-C (mmol/L) | 0.179 | 0.084, 0.383 | <0.001 |
| AST (U/L) | 1.005 | 0.997, 1.012 | 0.208 |
| eGFR (ml/min) | 0.998 | 0.995, 1.001 | 0.207 |
| Age when first taking statin | 0.971 | 0.959, 0.984 | <0.001 |
| CoQ10 | 0.323 | 0.157, 0.668 | 0.003 |
| CoQ10 daily dosage (mg) | 0.999 | 0.994, 1.004 | 0.72 |
| CoQ10 daily dosage per kilogram of body weight (mg/kg) | 0.991 | 0.973, 1.010 | 0.341 |
| CoQ10 daily dosage per body surface area (mg/m^2^) | 1.000 | 0.999, 1.000 | 0.456 |

AST: aspartate aminotransferase, BMI: body mass index, CI: Confidence Interval, CoQ10: coenzyme Q10, DBP: diastolic blood pressure, eGFR: estimated glomerular filtration rate, HbA1c: glycohemoglobin, HDL-C: high-density lipoprotein cholesterol, LDL-C: low-density lipoprotein cholesterol, OR: Odds Ratio, PIR: poverty income ratio, SBP: systolic blood pressure, TC: total cholesterol, TG: triglycerides. n represents the number of participants in the study sample, while N represents the weighted number of participants based on NHANES sampling principles. Continuous variables are presented as median (Q25, Q75). Categorical variables are presented as numbers (percentages).

**Supplementary Table 3** Multifactor Logistics regression results for two sets of models for CoQ10.

|  | **Characteristic** | **OR** | **95% CI** | ***P* value** |
| --- | --- | --- | --- | --- |
| Model 1 | CoQ10 | 0.344 | 0.160, 0.737 | 0.006 |
|  | Age | 1.022 | 1.008, 1.037 | 0.002 |
|  | Gender (male) | 1.662 | 1.148, 2.408 | 0.008 |
|  | Race |  |  |  |
|  | Mexican American | — | — |  |
|  | Other Hispanic | 1.932 | 0.811, 4.605 | 0.136 |
|  | Non-Hispanic White | 1.700 | 0.911, 3.173 | 0.095 |
|  | Non-Hispanic Black | 1.828 | 0.968, 3.452 | 0.063 |
|  | Other/multiracial | 1.959 | 0.961, 3.994 | 0.064 |
|  | Educational attainment |  |  |  |
|  | Below high school | — | — |  |
|  | High school | 1.157 | 0.734, 1.824 | 0.528 |
|  | Above high school | 1.065 | 0.626, 1.810 | 0.815 |
|  | PIR | 1.060 | 0.935, 1.203 | 0.358 |
| Model 2 | CoQ10 | 0.232 | 0.057, 0.942 | 0.041 |
|  | Age | 1.141 | 1.106, 1.177 | <0.001 |
|  | Gender (male) | 1.260 | 0.637, 2.492 | 0.503 |
|  | Race |  |  |  |
|  | Mexican American | — | — |  |
|  | Other Hispanic | 1.715 | 0.458, 6.420 | 0.421 |
|  | Non-Hispanic White | 0.877 | 0.393, 1.956 | 0.746 |
|  | Non-Hispanic Black | 1.238 | 0.479, 3.201 | 0.657 |
|  | Other/multiracial | 1.598 | 0.473, 5.395 | 0.447 |
|  | Educational attainment |  |  |  |
|  | Below high school | — | — |  |
|  | High school | 2.592 | 1.084, 6.195 | 0.032 |
|  | Above high school | 1.738 | 0.692, 4.363 | 0.237 |
|  | PIR | 1.135 | 0.947, 1.361 | 0.168 |
|  | Waist circumference | 1.017 | 0.999, 1.036 | 0.068 |
|  | Hypertension | 2.293 | 0.804, 6.535 | 0.120 |
|  | CVD | 1.309 | 0.487, 3.518 | 0.591 |
|  | Heart Failure | 1.618 | 0.719, 3.642 | 0.243 |
|  | LDL-C | 0.633 | 0.397, 1.011 | 0.055 |
|  | Age when first taking statin | 0.861 | 0.834, 0.888 | <0.001 |

CI: Confidence Interval, CoQ10: coenzyme Q10, CVD: cardiovascular disease, LDL-C: low-density lipoprotein cholesterol, OR: Odds Ratio, PIR: poverty impact ratio.

**Supplementary Table 4** Results of multicollinearity assessment for coq10 and nod logistic regression.

| **Characteristic** | VIF value |
| --- | --- |
| CoQ10 | 1.356915 |
| Age | 4.009753 |
| Gender (Male) | 2.004245 |
| Race |  |
| Other Hispanic | 2.354875 |
| Non-Hispanic White | 4.555558 |
| Non-Hispanic Black | 3.825725 |
| Other/multiracial | 3.581602 |
| Education attainment |  |
| High school | 3.674316 |
| Above high school | 3.860086 |
| PIR | 2.263565 |
| Hypertension | 2.185125 |
| CVD | 2.639633 |
| Heart failure | 2.075325 |
| Waist circumference | 3.592133 |
| LDL-C | 2.969639 |
| Age of first take statin | 4.210159 |

CI: Confidence Interval, CoQ10: coenzyme Q10, CVD: cardiovascular disease, LDL-C: low-density lipoprotein cholesterol, PIR: poverty impact ratio, VIF: Variance Inflation Factor.

**Supplementary Table 5** Results of the interaction between coq10 and NOD correlation

| **Characteristic** | OR | 95%CI lower | 95%CI upper | P value |
| --- | --- | --- | --- | --- |
| CoQ10*Gender(male) | 1.782 | 0.112 | 28.238 | 0.683 |
| CoQ10*CVD | 0.453 | 0.031 | 6.674 | 0.565 |
| CoQ10*Waist circumference | 1.022 | 0.969 | 1.077 | 0.422 |
| CoQ10*BMI | 1.099 | 0.907 | 1.332 | 0.336 |
| CoQ10*Hypertension | 0.430 | 0.022 | 8.384 | 0.579 |
| CoQ10*Heart failure | 0.000 | 0.000 | 0.000 | <0.001 |
| CoQ10*LDL-C | 0.521 | 0.052 | 5.258 | 0.581 |

BMI: Body mass index, CI: Confidence Interval, CoQ10: coenzyme Q10, CVD: cardiovascular disease, LDL-C: low-density lipoprotein cholesterol, OR: Odds Ratio, PIR: poverty income ratio.


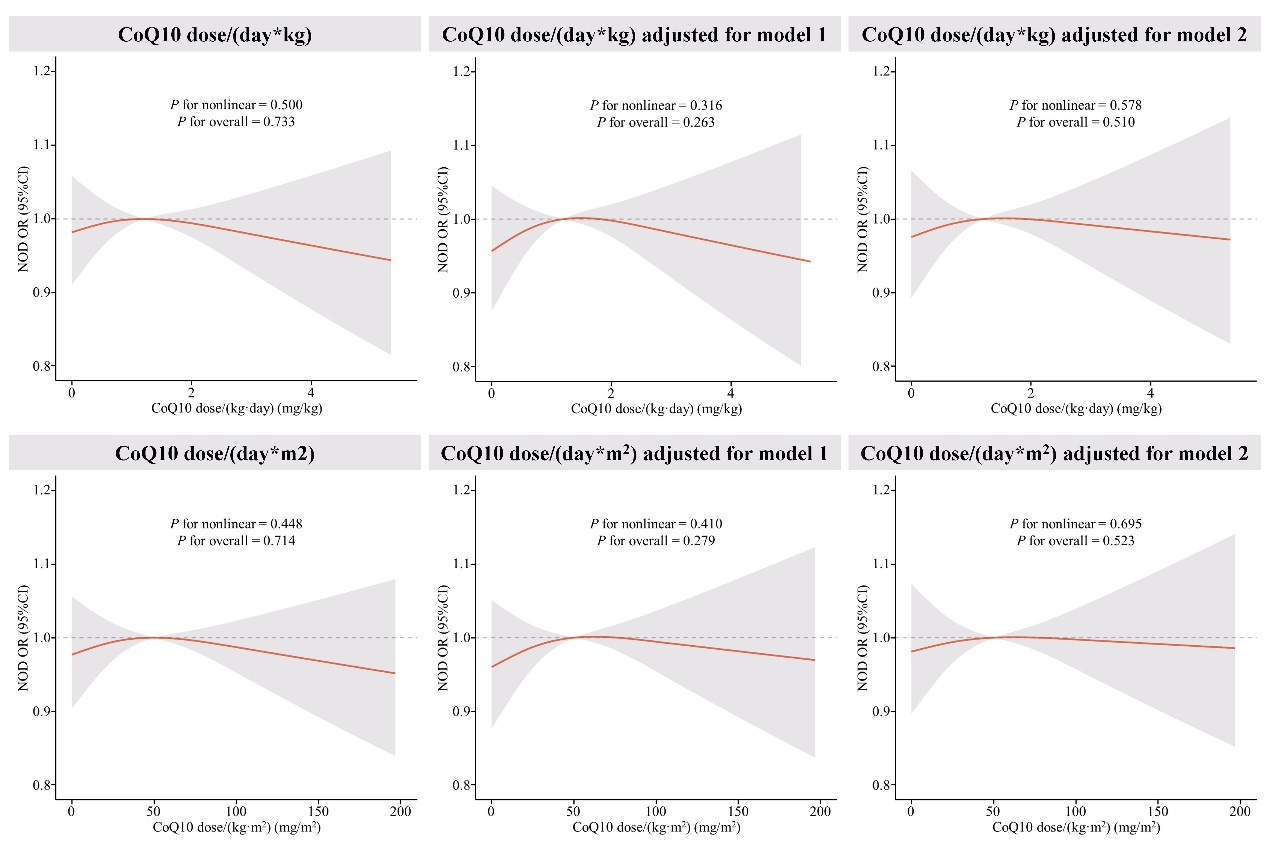
**Supplementary Fig. 1** RCS plot of the relationship between daily CoQ10 and new-onset diabetes per unit of body weight and per unit of body surface area.

Red lines represent odds ratios and shading indicates 95% confidence intervals. New onset diabetes was derived via logistics regression using RCS, following adjustments by model 1 and model 2. Model 1 adjusts for age, sex, race, PIR, and educational attainment. Model 2 further adjusts for hypertension, CVD, heart failure, waist circumference, LDL-C, and the age at which statin therapy was initiated.
